# Supplementary material for: Mechanisms governing the pioneering and redistribution capabilities of the non-classical pioneer PU.1
Source: Nat Commun. 2020 Jan 21;11:402. doi: 10.1038/s41467-019-13960-2 (PMC6972792; doi:10.1038/s41467-019-13960-2)
Supplement: Supplementary file 7 — Source data [file 41467_2019_13960_MOESM7_ESM.zip › Source_Data/FigureS2/FigureS2B_MotifScanOutput/KmeansCluster6/homerResults/motif26.similar.html]

motif26

## Information for motif26

G
A
T
C
C
T
G
A
C
T
G
A
A
G
T
C
A
G
T
C
C
G
T
A
A
T
G
C
C
G
T
A
  
Reverse Opposite:  

G
C
A
T
T
A
C
G
A
C
G
T
A
C
T
G
A
C
T
G
A
G
C
T
G
A
C
T
C
T
A
G
  

|  |  |
| --- | --- |
| p-value: | 1e-42 |
| log p-value: | -9.831e+01 |
| Information Content per bp: | 1.768 |
| Number of Target Sequences with motif | 648.0 |
| Percentage of Target Sequences with motif | 19.68% |
| Number of Background Sequences with motif | 4758.7 |
| Percentage of Background Sequences with motif | 11.06% |
| Average Position of motif in Targets | 100.1 +/- 53.0bp |
| Average Position of motif in Background | 99.6 +/- 67.4bp |
| Strand Bias (log2 ratio + to - strand density) | -0.1 |
| Multiplicity (# of sites on avg that occur together) | 1.09 |
| Motif File: | file (matrix) reverse opposite |

### Similar de novo motifs found

|  |  |  |  |  |  |  |  |
| --- | --- | --- | --- | --- | --- | --- | --- |
| Rank | Match Score | Redundant Motif | P-value | log P-value | % of Targets | % of Background | Motif file |
| 1 | 0.773 | T G A C A G C T A C T G C T A G A C G T A C T G A C T G A G C T C G A T | 1e-37 | -86.947599 | 6.53% | 2.22% | motif file (matrix) |
| 2 | 0.960 | A G C T A C T G A C G T A C T G A C T G A G C T G C A T C G T A T C A G | 1e-37 | -86.775887 | 21.74% | 13.18% | motif file (matrix) |
| 3 | 0.971 | C G T A C T G A A G T C A G T C C G T A A G T C T G C A | 1e-35 | -80.738733 | 17.89% | 10.36% | motif file (matrix) |
| 4 | 0.884 | A G T C A G C T A C T G C G A T C T A G A C T G G A C T C G A T C A G T A G T C | 1e-22 | -50.915529 | 5.83% | 2.54% | motif file (matrix) |
| 5 | 0.812 | T C A G C G A T A G T C G C A T T G C A T G C A G A T C G T A C G T C A A G T C C G T A | 1e-19 | -44.933648 | 3.31% | 1.12% | motif file (matrix) |
| 6 | 0.854 | C G A T A T C G A C G T A C T G A C T G A G C T A G C T C T G A C T A G C T G A | 1e-18 | -43.418498 | 3.58% | 1.30% | motif file (matrix) |
